# NKp46^+^ natural killer cells develop an activated/memory-like phenotype and contribute to innate immunity against experimental filarial infection

**DOI:** 10.3389/fimmu.2022.969340

**Published:** 2022-09-27

**Authors:** Nicolas Pionnier, Julio Furlong-Silva, Stefano A. P. Colombo, Amy E. Marriott, Valerine C. Chunda, Bertrand L. Ndzeshang, Hanna Sjoberg, John Archer, Andrew Steven, Samuel Wanji, Mark J. Taylor, Joseph D. Turner

**Affiliations:** ^1^ Centre for Drugs and Diagnostics, Department of Parasitology, Liverpool School of Tropical Medicine, Liverpool, United Kingdom; ^2^ Centre for Bioscience, John Dalton Building, Faculty of Science and Engineering, Manchester Metropolitan University, Manchester, United Kingdom; ^3^ Parasite and Vector Biology Research Unit, Department of Microbiology and Parasitology, Faculty of Science, University of Buea, Buea, Cameroon; ^4^ Research Foundation for Tropical Diseases and the Environment (REFOTDE), Buea, Cameroon

**Keywords:** natural killer cells (NK cells), lymphatic filariasis, *Brugia malayi*, innate lymphoid cells (ILC), eosinophils, Rag2 knockout (KO) mouse, neutrophils

## Abstract

Lymphatic filariasis and onchocerciasis are major neglected tropical diseases affecting over 90 million people worldwide with painful and profoundly disfiguring pathologies (such as lymphoedema or blindness). Type 2 inflammation is a hallmark of filarial nematode tissue infection and is implicated both in eosinophil dependent immunity and lymphatic or ocular immunopathologies. Type-2 innate lymphoid cells (ILC2) are known to play an important role in the initiation of type 2 inflammation in helminth infection. We therefore tracked comparative IL-12Rβ2^+^ ILC1, ST2^+^ ILC2 and NKp46^+^ natural killer (NK) innate lymphoid cell population expansions during *Brugia malayi* experimental peritoneal filarial infections using either immunocompetent or immunodeficient mice. In immunocompetent BALB/c animals, NKp46^+^ NK cells rapidly expanded representing over 90% of the ILC population in the first week of infection, whereas, surprisingly, ST2^+^ ILC2 failed to expand. NKp46^+^ NK cell expansions were confirmed in RAG2 deficient mice lacking adaptive immunity. Ablation of the NKp46^+^ NK cell compartment in RAG2 common gamma chain (gc) mice led to increased susceptibility to chronic adult *B. malayi* infection. This data was recapitulated using an *Onchocerca ochengi* male worm peritoneal implant model. When NKp46^+^ NK cells were depleted in RAG2 deficient mice using anti-NKp46 or asialo GM1 antibody injections over the first five weeks of *B. malayi* infection, susceptibility to adult *B. malayi* infection was significantly increased by 2-3 fold with concomitant impairment in eosinophil or neutrophil recruitments. Finally, we demonstrate that in RAG2 deficient mice, drug clearance of a primary adult *B. malayi* infection followed by challenge infection leads to resistance against early larval *B. malayi* establishment. This innate resistance is associated with bolstered NK and eosinophils whereby NKp46^+^ NK cells express markers of memory-like/enhanced activation (increased expression of interferon gamma and Ly6C). Our data promotes a novel functional role for NKp46^+^ NK cells in immunoprotection against experimental primary and secondary filarial infection which can proceed in the absence of adaptive immune regulation.

## Introduction

Lymphatic filariasis and onchocerciasis are parasitic helminth Neglected Tropical Diseases (NTDs) evoking severe morbidity. These filarial diseases are being prioritized for elimination. Despite encouraging results accomplished by mass drug administration programs over the last thirty years, the number of people infected with these filarial parasites remains significant in endemic regions (approximately 90 million combined) (1). The recent CoVID-19 pandemic significantly impacted on preventive chemotherapy treatment coverage and highlights the vulnerability of this long-term elimination strategy with preventative chemotherapy (2).

Filarial nematodes induce polarized host type 2 immune responses in experimental models which are associated with killing of developing larvae and induction of immunopathology in the lymphatics or ocular tissues (3, 4). Clinically, type-2 immune responses are also evident and associated with pathology, albeit as part of a more heterogeneous immune response including induction of regulatory immune networks and classical inflammation evoked by *Wolbachia* endobacteria and secondary microbial skin infections (5–7). Cellular mechanisms of type-2 anti-filarial responses include the proliferation of non-classical ‘alternatively activated’ polarized macrophages at the site of infection (8, 9), driving a profound chemotactic eosinophil recruitment from the blood (10–13) which in turn leads to granulocyte-dependent larvicidal effector response and granuloma formation (14–17). However, little is known on the pathogen sensing and sources of type-2 priming signals involved in the early stages of the infection. We have recently identified a clear IL-4/IL-13/IL-4Rα dependent mechanism for tissue macrophages proliferation and activation (13). However, we have also demonstrated that there are IL-4Rα-independent/IL-5- and CCR3-dependent pathways mechanisms that can also lead to eosinophil-dependent effector responses prior to patency (12), thus indicating that other early sources of Type 2 associated cytokines and ligands might be involved in this mechanism.

Innate lymphoid cells (ILCs) were first described as innate counterparts of T lymphocytes, being able to produce an array of effector cytokines but lacking adaptive antigen receptors (18, 19). Amongst these, type 2 ILCs (ILC2) are known for producing IL-4, -5, -9 and/or -13 in the context of helminth infection (20, 21) which promote CD4^+^ T-helper (Th)2 differentiation (22). In addition, these cells have also been reported to proliferate in sterile tissue sites following filarial nematode infection (20). Recent studies have shown using RAG2^-/-^γc^-/-^ immunodeficient animals (lacking functional lymphocytes and innate lymphoid cells) that chronic susceptibility to certain filarial infections is dependent both on lack of adaptive immune function and ablation of the ILC compartment (23–25). Beyond ILC2s, NK cells, which belong to the ILC1 family, have also been shown to produce IL-4 and IL-5 cytokines against *Brugia malayi* microfilaria (26) and contribute to the production of IL-13 in the context of a *Trichuris muris* infection (27). Furthermore, NK cells depletion led to increased *Litomosoides sigmodontis* filarial worm load (28).

Our present study focused on the exploration of ST2^+^ ILC2, IL-12Rβ2^+^ ILC1 (non NK) and NKp46^+^ NK cell populations dynamics and protective immune functional roles in the context of the human lymphatic filaria, *B. malayi*, using experimental infections of immunocompetent, RAG2^-/-^ and RAG2^-/-^γc^-/-^ immunodeficient animals and *via* antibody mediated cell depletions.

## Materials and methods

### Animals, infections, and treatments

BALB/c and BALB/c RAG2^-/-^γc^-/-^ male mice were purchased from Charles River UK whilst BALB/c RAG2^-/-^ mice were kindly provided by Prof Andrew McKenzie (MRC Laboratory of Molecular Biology, Cambridge University, UK) and by Prof. Dr. Antonius Rolink (Developmental and Molecular Immunology Department of Biomedicine, University of Basel, Switzerland). Mongolian gerbils *(Meriones unguiculatus*) were originally purchased from Charles River, Europe. BALB/c and RAG2^-/-^ mice and gerbils were subsequently bred in house. All animals were maintained in SPF conditions at the Biological Services Unit of the University of Liverpool. The experimental life cycle of *B. malayi* was maintained by passage between intraperitoneal infections of male gerbils and membrane-feeding of the Liverpool *Aedes aegypti* mosquitoes strain, as previously described to provide infectious stage L3 larvae for infections (13, 29).

Groups of between four and six male mice of 50-60 days of age (18-20g) were experimentally infected with 50 *B. malayi* L3 in a maximum volume of 200µL of RPMI medium and inoculated *via* intraperitoneal injection as previously described (13). Mice were euthanized by UK Home Office approved schedule 1 method at given time points (days or weeks) post-infection. All experiments on animals were approved by the ethical committees of LSTM and the University of Liverpool and were conducted according to Home Office Legislation and ARRIVE guidelines.

In NK depletion studies, mouse groups were treated through serial IP injections with either 0.5 mg anti-NKp46 antibody (clone 9E2, Biolegend) or 0.5 mg of its isotype control (mouse IgG1, Biolegend) per mouse every 6 days until 5 weeks post-infection. Alternatively, mouse groups were also subjected to serial IP injections with 50 µL of anti-Asialo-GM1 antibody (clone Poly21460, Biolegend) or its rabbit polyclonal IgG isotype control (Biolegend) every 4 days until 5 weeks post-infection.

In immune-primed studies, *B. malayi* infected mice were subcutaneously injected with flubendazole (Sigma) at 10 mg/kg 4 weeks post-infection. According to published protocols (30–32) a worm infection clearance/drug washout period of 5 weeks was considered prior to mice enrolling another *B. malayi* infection challenge as described in the previous paragraph.

For *Onchocerca ochengi* infections CB.17 SCID (5-6 weeks of age, from Charles River Europe), RAG2^-/-^ and RAG2^-/-^γc^-/-^ male mice were shipped to the Research Foundation for Tropical Diseases and the Environment (REFOTDE), Buea, Cameroon maintained in individually ventilated caging (IVC) with high-efficiency particulate air (HEPA) filtered air system (Tecniplast, UK). Mice were infected with intraperitoneal *O. ochengi* adult worms implantations as previously described (29, 30) and culled at 6 weeks post-infection. Parasites and peritoneal cell exudates were recovered and analyzed as described below. All experiments carried out in Cameroon were approved by the Animal Care Committee, REFOTDE.

### Parasites and peritoneal exudate cell isolation

Motile *B. malayi* or *O. ochengi* parasites and peritoneal exudate cells (PECs) were recovered by peritoneal lavage with 10mL PBS +5% FCS at necropsy and worms were enumerated by microscopy. After parasite enumeration and removal, peritoneal exudates were centrifuged (250G, 5min, 4°C) and cells were resuspended in 1 mL FACS buffer (PBS, 5% FCS, 1mM EDTA). Total cell counts were performed in PBS/0.04% trypan blue (Sigma) using a hemocytometer (KOVA^®^ Glasstic Slide). Cell infiltrates were consequently phenotypically analyzed through flow cytometry.

### Flow cytometry

Proportions of granulocytes (eosinophils, neutrophils) populations were determined by flow cytometry using anti-SiglecF-PE (BD, clone E50-2440) and anti-Ly6G-BV650 (Biolegend, clone 1A8) rat anti-mouse antibodies. For innate lymphoid cell populations characterization, a lineage cocktail comprised of anti-CD8 (eBioscience, clone 53-6.7), anti-B220 (eBioscience, clone RA3-6B2), anti-F4/80 (eBioscience, clone BM8), anti-SiglecF (Miltenyi Biotec, clone ES22-10D8), anti-CD4 (eBioscience, clone GK1.5), anti-Ly6G (eBioscience, clone RB6-8C5) and anti- FcƐR1 (eBioscience, clone MAR-1) antibodies conjugated to APC was used in combination with anti-ST2-PE (eBioscience, clone RMST2-2), anti-NKp46-AF700 (BD, clone 29A1.4), anti- IL-12Rβ2-AF488 (R&D, clone 305719) and anti-CD127-PerCPCy5.5 (eBioscience, clone A7R34). In addition, NK cell activation was investigated using additional anti-IFNγ- BV510 (clone XMG1.2, Biolegend) and anti-Ly6C-BV785 (clone HK1.4, Biolegend) antibodies. All panels included a viability staining using the eF450 viability dye (Invitrogen) and all antibodies were used at a 1/40 dilution. Representative gating strategy is given in **Supplementary Figure 1**. Flow cytometric acquisition was performed on a BD LSR II and data analyzed on FlowJo Software (version 10.0.7).

### Statistical analysis

Statistical analysis was carried out using GraphPad Prism v8. Sample size, normality (Shapiro-Wilk test) and homoscedasticity (Bartlett’s test) were tested prior to further analysis. Data from 2-3 separate experiments were pooled where possible, i.e., if variances are not significantly different. Significant differences between groups were evaluated by either unpaired T test (2 groups) or Mann-Whitney (>2 groups) or Kruskal-Wallis with Dunn’s *post-hoc* tests (>2 groups). Significance was defined as *: p < 0.05; **: p < 0.01; ***: p < 0.001 and ****: p < 0.0001.

## Results

### NKp46^+^ NK cells are the major ILC population at the site of *Brugia malayi* infection

**Figure 1:**
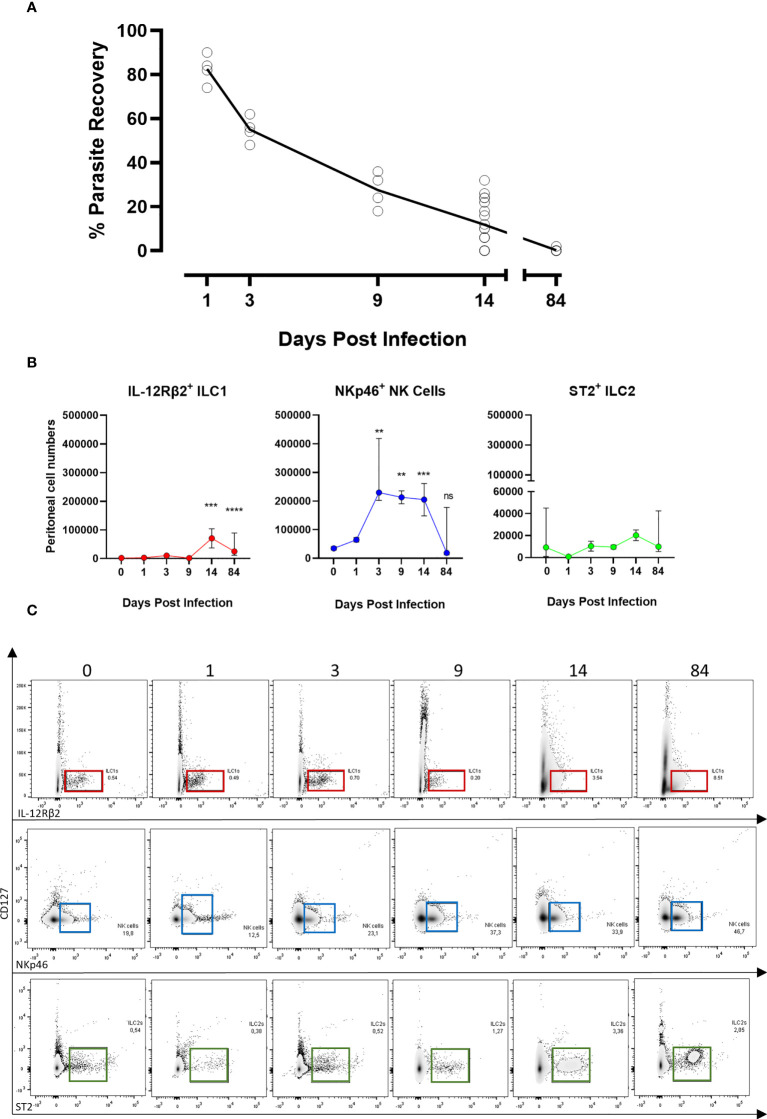
ILC populations dynamics at the site of *Brugia malayi* infection. **(A)** Parasite recovery in BALB/c infected mice over the time. **(B)** IL-12Rβ2^+^ ILC1s (red), NKp46^+^ NK (blue) and ST2^+^ ILC2s (green) cell populations expansion in the peritoneal cavity of BALB/c immunocompetent mice following *B. malayi* infection. Kruskal-Wallis tests followed by Dunn’s multiple comparisons tests, n=4-25, pooled experiments. Significance is given as **:p < 0.01, ***:p < 0.001 and ****:p < 0.0001. ns, not significant. **(C)** Representative density plots and gating from lineage cocktail negative cells for IL-12Rβ2^+^ ILC1s (red), NKp46^+^ NK cells (blue) and ST2^+^ ILC2s (green) using IL-12Rβ2, NKp46 and ST2 respective pan markers between 0 and 84 days post-infection.

As prior observed (12, 13), in BALB/c immunocompetent animals, parasite recovery drastically declined within the first two weeks post-*B. malayi* infection (approx. 30% at day 9 and 10% at day 14, **Figure 1A**) and mice completely cleared infection before the chronic patent time-point (0% recovery at week 12, **Figure 1A**). As ILCs can promote the initial immune response to nematode parasites (33, 34), we examined the relative expansions of ILC populations at the peritoneal site of *B. malayi* infection. Surprisingly, we did not observe significant expansion of ST2^+^ ILC2s at the site of infection within the first 14 days post-infection (**Figures 1B, C**). In regard to IL-12Rβ2^+^ ILC1s, a significant increase was apparent between D14 and D84 (**Figures 1B, C**). In contrast, NKp46^+^ NK cells represented the predominant ILC population in the peritoneal cavity of either naïve or infected mice at any time point post-infection (**Figures 1B, C**).

### NKp46^+^ NK cell impairment through γc deficiency is associated with increased susceptibility to filarial parasites

**Figure 2:**
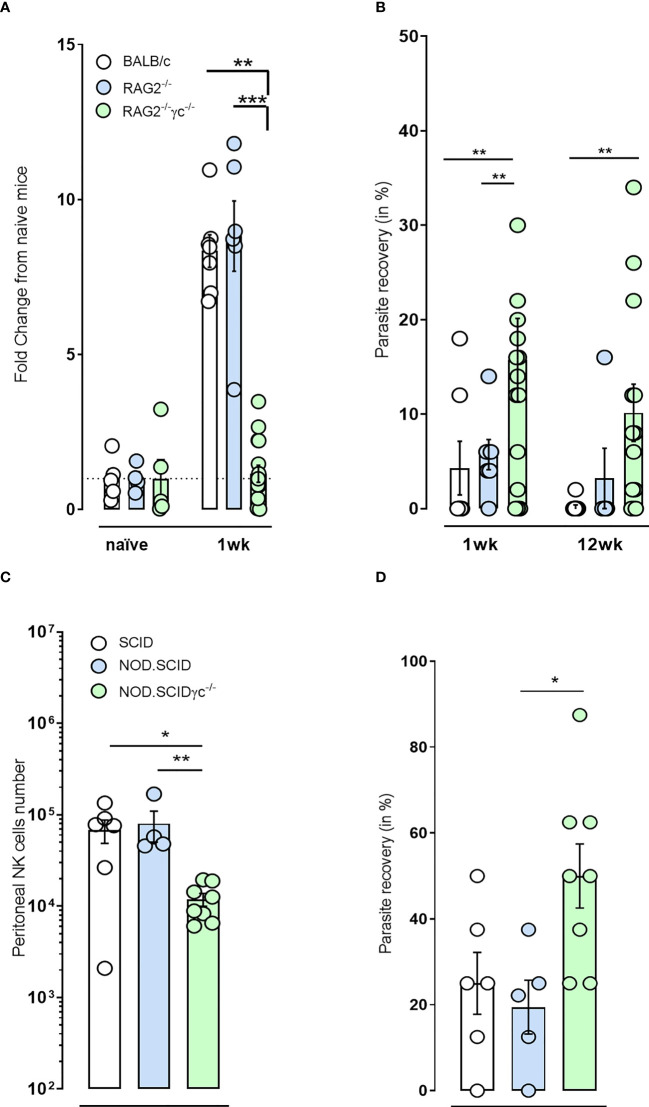
NKp46^+^ NK cell impairment in immunodeficient mice *via* γc deficiency renders mice more susceptible to *B. malayi and Onchocerca ochengi* infection. **(A)** Temporal changes in NKp46^+^ NK cell populations at the site of infection or **(B)** Parasite recovery in infected BALB/c immunocompetent, RAG2^-/-^ and RAG2^-/-^γc^-/-^ immunodeficient mice at either 1 or 12 weeks post- *B. malayi* infection. **(C)** NKp46^+^ NK cell numbers at the site of infection and **(D)** Parasite recovery in infected SCID, NOD.SCID and NOD.SCIDγ^-/-^ immunodeficient mice 6 weeks post- *O. ochengi* worm infection. Statistical significance is derived from a Kruskal-Wallis test followed by Dunn’s multiple comparisons tests, n=5-16, two pooled experiments *(B. malayi*, 1wpi repeated) n=4-8, single experiment *(O. ochengi*). Significance is given as: *:p < 0.05, **:p < 0.01, ***:p < 0.001.

We infected RAG2^-/-^ and RAG2^-/-^γc^-/-^ immunodeficient and BALB/c immunocompetent mice with *B. malayi* infectious larvae and examined impact on NKp46^+^ NK cell expansion within the peritoneal infection site. NKp46^+^ NK cell expansion was intact and similar to WT mice after one-week infection in RAG2^-/-^ mice. However, the additional ablation of γc cytokine signaling inhibited the typical expansion of NKp46^+^ NK cells in the peritoneal cavity (**Figure 2A**). BALB/c mice rapidly eliminated the parasite (4% and 1% parasite recoveries at 1 and 12 weeks p.i., respectively, **Figure 2B**). A similar trend was observed for the RAG2 deficiency with only 20% (1/5) of the mice retaining adult *B. malayi* parasites by 12 week p.i. despite 86% positive infection (6/7) at 1 week p.i. (**Figure 2B**). Combined RAG2 and γc impaired mice were significantly more susceptible to *B. malayi* larvae at one week compared with either WT or single RAG2 knockout mice (**Figure 2B**) and the majority remained susceptible to adult infections at 12 weeks p.i. (10/13, 77%, p=0.01).

In addition, reduced numbers of NKp46^+^ NK cells were also observed following implantation of adult male *Onchocerca ochengi* into the peritoneal cavity in NOD.SCID γc^-/-^ mice (NSG mice) compared with either CB.17 or NOD.SCID mice (**Figure 2C**). This was associated with an increased rate of survival of *O. ochengi* in NSG mice (**Figure 2D**). These data support a hypothesis that ILC and in particular NKp46^+^ NK cells may have a functional role in innate resistance to filarial parasites.

### NKp46^+^ NK cell depletion in RAG2^-/-^ mice leads to increased filarial parasite susceptibility and impaired granulocyte recruitment to the site of infection

**Figure 3:**
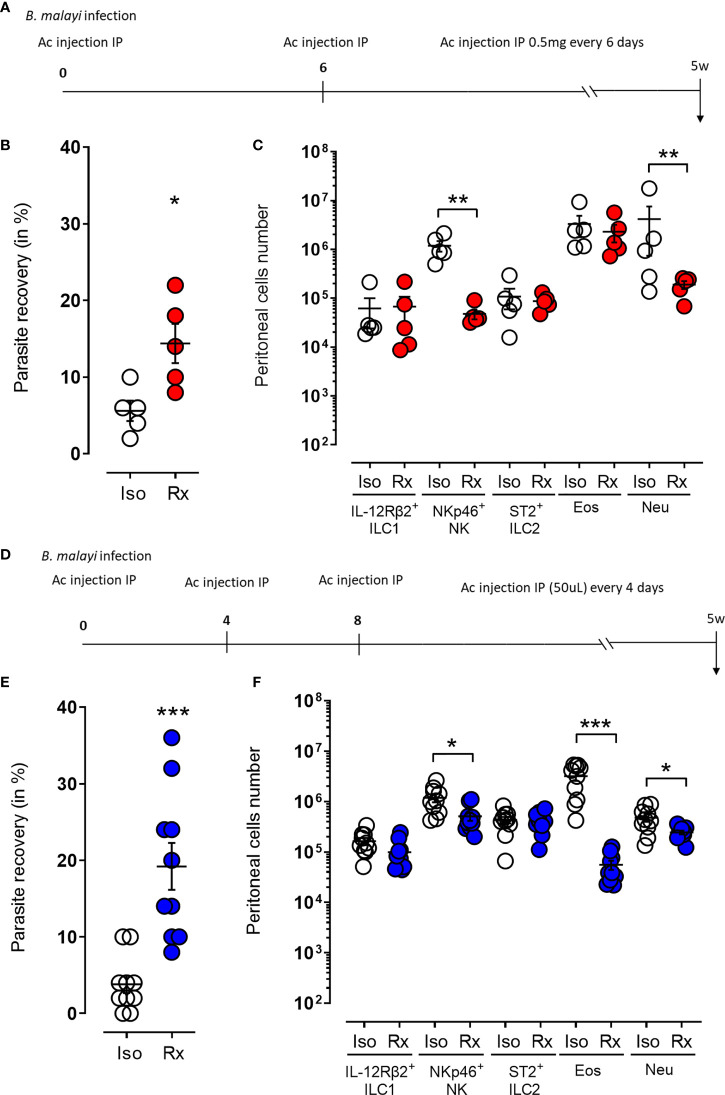
Temporal ablation of NKp46^+^ NK cells in RAG2^-/-^ mice leads to increased susceptibility to *B. malayi* and impaired granulocytes recruitment to the site of infection. **(A)** Schematic representation of the study design where *B. malayi* infected RAG2^-/-^ mice were intraperitoneally (IP) injected with 0,5mg anti-NKp46 antibody or its isotype control every 6 days until 5 weeks post-infection. **(B)** Parasite recovery in isotype control treated mice (Iso) or anti-NKp46 treated mice (Rx) at 5 weeks post-infection. **(C)** Innate lymphoid cells (IL-12Rβ2^+^ ILC1, NKp46^+^ NK, ST2^+^ ILC2) and granulocytes (eosinophils – Eos and neutrophils – Neu) numbers in the peritoneal cavity of control (Iso) or anti-NKp46 treated (Rx) infected mice at 5 weeks post-infection. **(D–F)** similar study in which *B. malayi* infected RAG2^-/-^ mice were IP injected with 50µL of anti-asialoGM1 antibody or its isotype control every 4 days until 5 weeks post-infection. Respective parasite recovery is given in **(E)** and peritoneal cell numbers in **(F)**. Unpaired T-tests, n=5-10, single experiment. Significance is given as *:p < 0.05, **:p < 0.01 and ***:p < 0.001.

As the γc depletion does not only prevent NK cell and other ILC development but also impinges multiple cytokine signaling pathways, we depleted NKp46^+^ NK cells in BALB/c RAG2^-/-^ mice using either anti-NKp46 or an anti-asialoGM1 monoclonal antibodies, both widely used in the context of NK cell depletion (35–37). We selected BALB/c RAG2^-/-^ mice based on our observations that these lymphopenic mice had sufficient residual γc- dependent innate immune mechanisms to mediate resistance to long-term *B. malayi* and in order reduce redundancy of NK-independent adaptive immune pathways intact within WT mice. When RAG2^-/-^ mice were treated with regular injections of the anti-NKp46 antibody (**Figures 3A–C**), a significant increase in parasite recovery was observed at 5 weeks post-treatment (14.4% *vs*. 5.6% respectively, p=0.05 **Figure 3B**). This was linked to a significant decrease in NKp46^+^ NK cell numbers (p=0.01, **Figure 3C**) as well as a statistically significant reduction in neutrophil numbers (p=0.01, **Figure 3C**). Consistent with these observations, a similar outcome was obtained by treating infected RAG2^-/-^ mice with regular anti-asialoGM1 antibody (**Figures 3D–F**). This regime resulted in a significant difference in parasite recovery at 5 weeks post-treatment (19.2% *vs.* 3.8% respectively, p=0.001 **Figure 3E**) associated with a significant reduction in NKp46^+^ NK cell, eosinophil and neutrophil numbers (p=0.05, p=0.01 and p=0.05 respectively, **Figure 3F**). Interestingly, a similar NK depletion performed over the first week of infection had no significant effect on parasite recovery or granulocyte recruitment (**Supp Figure 2**) which might indicate that a sustained expanded NK cell population over a prolonged larval development phase of *B. malayi* could be required to mediate significant anti-filarial immunity.

### A NKp46^+^ NK cell enhanced activation/memory phenotype is apparent in immune-primed challenged mice

**Figure 4:**
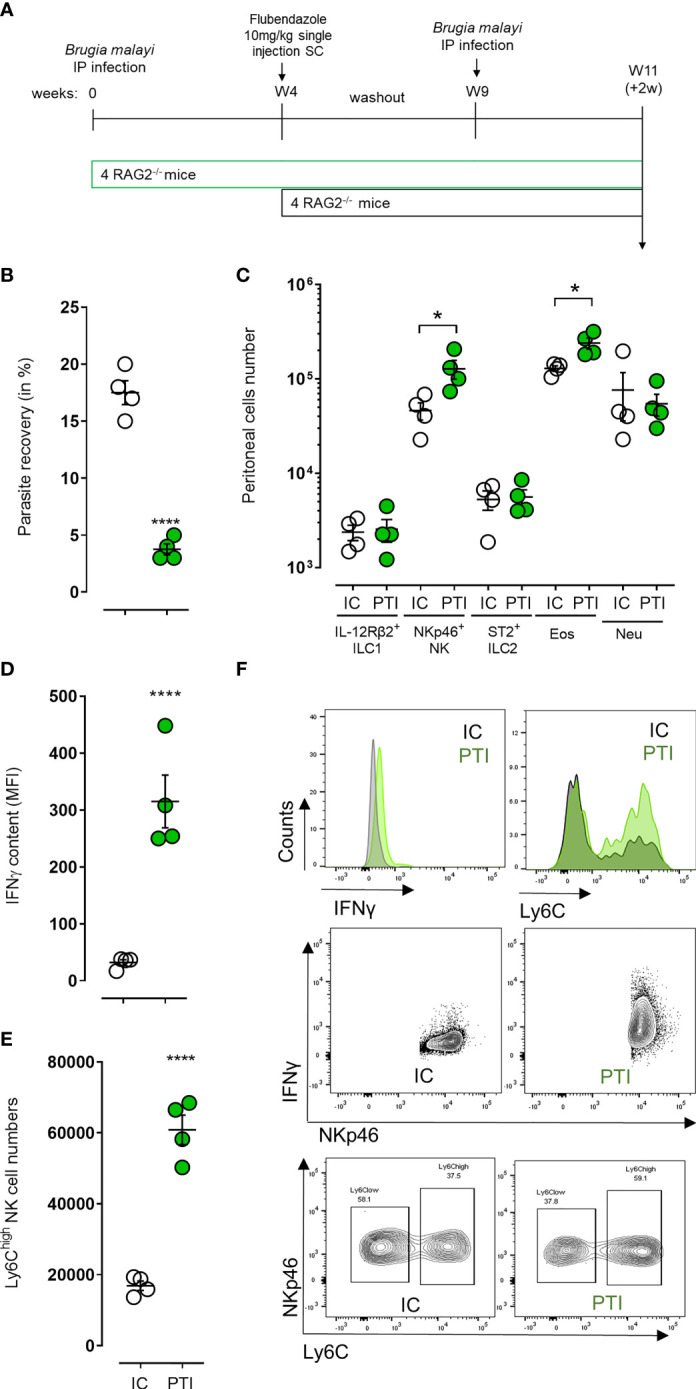
NKp46^+^ NK cells display signs of enhanced/memory-like activation and boost anti-filarial immune response in challenge after immune-priming. **(A)** Schematic representation of the study design where *B. malayi* infected RAG2^-/-^ mice were treated with flubendazole at 10mg/kg 4 weeks post-infection to clear them out of remaining worms before being challenged again at week 9 with *B. malayi* parasites and a readout 2 weeks post-challenge. **(B)** Parasite recovery in infected control mice (IC, clear) or primed treated infected (PTI, green) mice 2 weeks post-challenge. **(C)** Innate lymphoid cells (IL-12Rβ2^+^ ILC1, NKp46^+^ NK, ST2^+^ ILC2) and granulocytes (eosinophils – Eos and neutrophils – Neu) numbers in the peritoneal cavity of infected control (IC, clear) or primed treated infected (PTI, green) mice at 2 weeks post-challenge. **(D)** IFNγ content expressed as MFI in peritoneal NKp46^+^ NK cells from IC (clear) or PTI (green) mice 2 weeks post-challenge. **(E)** Ly6C^high^ NKp46^+^ NK cell numbers in the peritoneal cavity of IC (clear) or PTI (green) mice at readout. **(F)** Representative histograms and flow plots for IFN and Ly6C expression or cell counts on either pre-gated NK cells or depending on NKp46 expression in IC and PTI mice at readout. Unpaired T-tests, n=4, single experiment. Significance is given as *:p < 0.05 and ****:p < 0.0001.

A memory-like phenotypic response can be elicited by a variety of innate immune cells when the immune system is primed either *in vivo* or *in vitro* (38). In addition, there is evidence that ILCs and especially NK cell-mediated immune responses share common features with adaptive immunity, and that these cells acquire immunological memory in a similar manner to T and B cells (39, 40). We thus investigated the activation and memory-like properties of NKp46^+^ NK cells in an immune-primed context where 4-week *B. malayi*-infected mice were treated with flubendazole at 10mg/kg to clear infections before being re-infected 5 weeks later with *B. malayi* larvae and culled 2 weeks post-infection (**Figure 4A**). Secondary infected mice were more resistant to *B. malayi* challenge (17.5% *vs*. 3.75%, p=0.0001 **Figure 4B**) and this was associated with a significant increase in eosinophil numbers (p=0.05, **Figure 4C**). Interestingly, this was also accompanied with increased peritoneal NKp46^+^ NK cell numbers (p=0.05, **Figure 4C**) displaying significant changes in activation markers such as an enriched IFNγ content (p=0.0001, **Figures 4D, F**) and a marked Ly6C^high^ phenotype (p=0.0001, **Figures 4E, F**).

## Discussion

Infection with filarial nematodes remains a major cause of global morbidity *via* induction of immunopathology. Further, chronic filarial nematode infection modulates immune responses in the context of co-infection, allergy, and autoimmune disease (41), yet our understanding of the fundamental cellular mechanisms of immunity during infection is limited. We have previously demonstrated, through the use of RAG-deficient and SCID mice, a pivotal role for the innate compartment in the immunological control of filarial parasite infection (13, 25). However, the relative contributions made by specific innate cell populations is poorly understood. The data presented here provides the first evidence of a major role for NKp46^+^ NK cells in protection against *B. malayi* and *O. ochengi* infection in immunocompromised animals. NKp46^+^ cells expanded rapidly during the early stages of the infection and their depletion, *via* the use of γc^-/-^ animals and neutralizing antibodies, rendered mice susceptible to chronic infection. We also demonstrated that these cells further expand and exhibit an activated/memory-like phenotype associated with an augmented innate immune protection against *B. malayi* challenge infection in the absence of adaptive immunity.

There is a well-established role for NK cells in the defense against infection by viral, bacterial, and protozoan organisms (42). In these contexts, NK cells act as rapid first responders, with key roles in the production of cytokines and the direct lysis of cells. However, their role in defense against macroparasites is poorly defined. In previous studies investigating permissiveness of filarial infection in immune-deficient mouse strains, it was noted that depleting NK cells resulted in reduced *B. malayi* burden in C57BL/6-*scid/scid* mice (43). Complementary to this was the observation that enhancing NK cell activation, *via* injection with the TLR agonist poly(I:C), increased the permissiveness of NOD/LtSz-*scid/scid* mice (which normally show low NK cell activity and complement deficiency) to *B. malayi* infection (43). This suggested that the level of NK cell activity positively correlated with the permissiveness of the host to infection. However, in a study using BALB/c mice infected with *L. sigmodontis*, NK cells (identified as Dx5^+^CD3^-^) expanded significantly from 35 days post infection in the pleural cavity and down-regulated the inhibitory receptors Ly6C, Ly6A and Ly6G2, suggesting they are functioning here as effector cells rather than immunoregulators (28). Furthermore, their depletion with anti-asialoGM antibody increased both adult and mf burdens indicating that NK cells have an anti-filarial effect. In addition, *ex vivo* culture of NK cells (identified as CD3^-^CD56^+^) with *B. malayi* mf and L3 resulted in a contact-dependent increase in expression of the activation markers CD69 and CD71 and the pro-inflammatory cytokines IFNγ and TNFα (26). These data indicate that NK cells respond to filarial infection by homing to the site of infection and increasing their activation state in a manner that is detrimental to the survival of the parasite. This is consistent with our data showing that depletion of NKp46^+^ NK cells is sufficient to increase susceptible to *B. malayi* infection in the absence of adaptive immunity. We expanded these observations by demonstrating NKp46^+^ NK cells developed an enhanced activation phenotype/memory-like capacity within the peritoneal cavity which was associated with innate immunological resistance against subsequent challenge following anthelmintic drug clearance of the primary infection. Such a phenomenon of ‘innate resistance’ has been prior described for challenge infection with *Onchocerca lienalis* skin mf infection following microfilaricidal drug clearance in lymphopenic SCID mice (44). This apparent NKp46^+^ cell-intrinsic memory-like phenotype and its contribution to the resistance to filarial infection requires further interrogation, using, for instance, adoptive transfer and recall activation assays.

The mechanism by which NKp46^+^ NK cells mediate parasite reduction remains unclear. In previous publications we have demonstrated that eosinophils are larvicidal effector cells during *B. malayi* infection, and that their IL-5-/CCR3-dependent recruitment is essential for parasite expulsion in immuno-competent BALB/c mice (12, 13). It was observed that in susceptible immunocompromised RAG2^-/-^γc^-/-^ mice, which lack a functional NK cell compartment as well as being deficient in T and B cells, the granulocyte population in the peritoneal cavity during *L. sigmodontis* infection skewed toward neutrophils when compared to immunocompetent mice in which eosinophils predominate (23). Since then models of adoptive immune cell transfer into RAG2IL-2Rγ-deficient C57BL/6 mice have been developed, allowing for further pin-pointing of cellular mechanisms of anti-filarial immunity (24). We observed that when NKp46^+^ NK cells were depleted using an anti-asialoGM1 antibody, eosinophil but also neutrophil recruitment to the peritoneal cavity was reduced. Thus, in the absence of an intact type-2 adaptive immune response, NKp46^+^ NK cells may facilitate filarial larval parasite killing by co-ordinating the recruitment and activation of eosinophil and/or neutrophil effector cells alongside with subsequent granuloma formation and extracellular traps release (17, 45, 46). However, with recent reports on the anti-asialoGM1 antibody also having off-target effects on *in vivo* basophil populations (47) and on the use of anti-NKp46 antibody resulting in blocked activation rather than cellular depletion (48), further investigations are needed to assess ILC activation in the context of these NK depletion studies. Regarding off-target effects of anti-asioloGM1 on basophils, we and others have demonstrated that eosinophils rather than basophils are the major effector cell-type mediating attrition of filarial infections in experimental models and that basophils play a negligible role in filarial immunity (12, 13, 49). Thus, it is doubtful that anti-asioloGM1 treatment is increasing susceptibility *via* and off-target depletion of this granulocyte population.

Beyond parasitic infection, there is a clear link between NK cell activity and regulation of eosinophilia during airway hypersensitivity reactions. In humans suffering from severe asthma, NK cell activation measured by expression of CD69 and NKG2D correlated strongly with blood eosinophilia (50). Consistent with a role for NK cells as drivers of the eosinophilic response, in mouse models of allergic inflammation sensitization challenge with ovalbumin (OVA) resulted in a concomitant increase in NK cells and eosinophils (51, 52). Further, when NK cells were depleted prior to sensitization, lung eosinophilia was impaired (51, 52). *In vitro* co-culture of NK cells and eosinophils demonstrated that activated NK cells are capable of triggering eosinophil degranulation (53). Interestingly, NK cells have also been shown to act as negative regulators of eosinophils, capable of triggering their apoptosis under *in vitro* co-culture conditions (50, 53). Further, depletion of NK cells using anti-asialoGM1 at the peak of OVA-induced airway inflammation delayed the resolution of lung eosinophilia (54). Thus, under type-2 inflammatory conditions, NK cells perform a dual function first recruiting eosinophils and then subsequently inducing their apoptosis to resolve inflammation. Therefore, it will be interesting to define the mode through which NKp46^+^ NK cells recruit eosinophils during filarial infection, whether this is direct, *via* the secretion of chemoattractant, or if they support the development of M2 macrophages known to be key recruiters of eosinophils during *B. malayi* infection (13). There is also some evidence that NK cells express IL-5 in response to contact with *B. malayi* mf (26). However, this data was derived from a single *ex vivo* co-culture model of NK cell-*B. malayi* mf interaction. Additionally, *B. malayi* L3 did not provoke an IL-5 response under the same conditions. Therefore, further work is required to determine whether NK cell-secreted IL-5 is a direct recruiter of eosinophils during *in vivo* infection.

Our data illustrated a lack of ST2^+^ ILC2 expansion in the peritoneal infection site following *B. malayi* infection, which was surprising given the predominant protective type-2 polarized immune response triggered in this filarial experimental infection model (12, 13). In murine models of small intestinal-dwelling nematodes, ILC2s are known to play a role in the priming of Th2 responses, functioning as potent producers of Th2 cytokines (55). Depletion of ILC2s results in a failure to expel *Nippostrongylus brasiliensis* (34, 56), and impairs IL-4-driven differentiation of Th2 CD4^+^ T cells following infection with *Heligmosomoides bakeri* (22). Further, priming of ILC2s in the gut, *via* infection with *H. bakeri* or *T. spiralis*, can trigger ILC2 migration to distal tissues such as the lung and enhances subsequent larval trapping during the lung migratory stage of *N. brasiliensis* (57, 58). However, the role of ILC2s during filarial nematode infection remains poorly understood. During *Loa* infection in humans, the frequencies of cKit^+^ILCs (ILC2s and ILC3s) and cKit^+^IL-13^+^ILCs (ILC2s) in peripheral blood were observed to increase (59). The cKit^+^ILCs expressed an array of TLRs and were capable of producing Th1, Th2, and Th17 cytokines upon stimulation by cytokine cocktail *ex vivo.* Interestingly, cKit^+^IL-13^+^ILC frequency did not correlate with the frequency of Th2 CD4^+^T cells suggesting that ILC2s might not be required for sustaining peripheral Th2 responses (59). However, the authors derived samples from already chronically infected individuals and therefore this data does not rule out a role for ILC2s in the early induction of Th2 CD4^+^T cell responses. Despite ST2 being expressed by a subset of ILC2s only (18), during chronic infection of *L. sigmodontis* infection in Balb/c mice, efficient clearance of *L. sigmodontis* microfilariae is ST2 (IL-33R)-dependent, which might implicate a role for ST2-dependent ILC2 expansion in the control of filarial microfilaremias (60). It has been demonstrated that IL-5 producing ILC2s expanded specifically in the pleural cavity infection site of *L. sigmodontis*, whereas in secondary lymphoid tissue no change in ILC2 frequency is observed (20). Further, the frequency of ILC2s in the blood reduces during *L. sigmodontis* infection (20), indicating that an increase in ILC2 frequency in the pleural cavity may be a consequence of recruitment from peripheral tissues. The ILC2 expansion following *L. sigmodontis* but not *B. malayi* infection in serous cavities might be explained by the *L. sigmodontis* larvae migration through the lymphatics and pulmonary blood vessels before the thoracic cavity invasion (61). Conversely, in our studies, *B. malayi* larvae are introduced directly into the peritoneal cavity. Considerable damages to the endothelium and lung parenchyma are evidenced as part of the *L. sigmodontis* larval migration route. Damaged tissues caused by nematode invasion are known to trigger a variety of ‘alarmin’ release from stromal cells, such as IL-33, which are key triggers of robust ILC2 expansion (62).

Consistent with our data however, ILC2s are not ubiquitously required for immune control of all nematode parasites. Whilst ILC2s respond strongly to small intestinal nematodes (22, 34, 56) they fail to expand during infection with the cecal dwelling *Trichuris muris* and their depletion has no effect on parasite expulsion (63). Thus, it seems that the role of ILC2s in nematode infection is highly contextual and further work is required to establish causality between ILC2 activation and immunity to filarial nematodes, especially considering the diversity of clinically and veterinary important filarial nematodes and variability in their migration routes and adult niches of parasitism.

The present study implicates a functional role for NKp46^+^ NK cells in innate anti-filarial immune-protection in the absence of adaptive immunity. This mechanism may be relevant in situations whereby adaptive immunity is impaired, anergized or actively suppressed (for instance genetic variability or chronic co-infection situations in humans). Further, our data provides a mechanistic rationale why additional common gamma-chain deficiency is a requirement for chronic susceptibility in lymphopenic mouse models of human/veterinary filarial pathogens.

Follow-up investigations are needed to demonstrate their functional interplay with effector cells such as eosinophils and the full repertoire of memory-like signals harbored by NKp46^+^ NK cells following a secondary infection which may exert a degree of innate-memory protection from challenge. Research on NK cell biology and plasticity is progressing and reveals a wider scope of NK cell functional fates than initially thought (18, 64). Selecting the relevant phenotypic markers will be therefore crucial to allow a comprehensive scrutinization of the relevant NK subsets and their dynamics following filarial primary or secondary infection.

## Data availability statement

The original contributions presented in the study are included in the article/**Supplementary Material**. Further inquiries can be directed to the corresponding author.

## Ethics statement

The animal study was reviewed and approved by the Liverpool School of Tropical Medicine ethical committee, the University of Liverpool ethical committee and the REFOTDE Animal Care Committee.

## Author contributions

Conceived and designed the analysis: NP, SW, MT, and JT. Collected the data: NP, JF-S, AM, VC, BN, HS, JA, and AS Contributed data or analysis tools: NP, JF-S, SC, AM, HS, SW, and JT. Performed the analysis: NP and JF-S. Wrote the paper: NP, JF-S, SC, and JT. All authors contributed to the article and approved the submitted version.

## Funding

This work was supported by a Bill and Melinda Gates Foundations Grand Challenges Explorations grant (OPP1119043) to JT, SW, and MT, and a Medical Research Council New Investigator Research Grant (MR/L018756/1) to JT. The funding bodies had no roles in the design of the study and collection, analysis, and interpretation of data. This work was supported, in whole or in part, by the Bill & Melinda Gates Foundation [OPP1119043]. Under the grant conditions of the Foundation, a Creative Commons Attribution 4.0 Generic License has already been assigned to the Author Accepted Manuscript version that might arise from this submission.

## Conflict of interest

The authors declare that the research was conducted in the absence of any commercial or financial relationships that could be construed as a potential conflict of interest.

## Publisher’s note

All claims expressed in this article are solely those of the authors and do not necessarily represent those of their affiliated organizations, or those of the publisher, the editors and the reviewers. Any product that may be evaluated in this article, or claim that may be made by its manufacturer, is not guaranteed or endorsed by the publisher.

## Supplementary material

Supplementary Figure 1 Flow cytometry gating strategy for leucocytes immunophenotyping. Doublets **(A)**, debris **(B)** and dead cells **(C)** were excluded and pan markers antibodies were utilised for the different cell populations. **(D)** Eosinophils and neutrophils were gated as SiglecF^+^ Ly6G^-^ and SiglecF^-^ Ly6G^+^ respectively. Innate lymphoid cells **(E–H)** were pre-gated on lineage negative cell populations **(E)** (CD8^-^, B220^-^, F4/80^-^, SiglecF^-^, CD4^-^, Ly6G^-^, FcγR1^-^) and identified as CD127^-/low^IL-12Rβ2^+^ST2^-^NKp46^-^ for ILC1s **(F)**, CD127^low^IL-12Rβ2^-^ST2^+^NKp46^-^ for ILC2s **(G)** and CD127^-/low^IL-12Rβ2^-^ST2^-^NKp46^+^ for NK cells **(H)**.

Supplementary Figure 2 Short-term temporal ablation of NKp46^+^ NK cells in RAG2^-/-^ mice is not sufficient to confer treated mice an increased susceptibility to *B. malayi* parasites. *B. malayi* infected RAG2^-/-^ mice were intraperitoneally injected with 0,5mg anti-NKp46 antibody or its isotype control in two occasions (at day 0 and day 3 post-infection) and culled at 1 week post-infection. **(A)** Parasite recovery in isotype control treated mice (Iso) or anti-NKp46 treated mice (Rx) at readout. **(B)** Innate lymphoid cells (IL-12Rβ2^+^ ILC1, NKp46^+^ NK, ST2^+^ ILC2) and granulocytes (eosinophils – Eos and neutrophils – Neu) numbers in the peritoneal cavity of control (Iso) or anti-NKp46 treated (Rx) infected mice at readout. Unpaired T-tests, **n=5**, single experiment. Significance is given as *: p <0.05.
