# Supplementary figures and images for: NKp46+ natural killer cells develop an activated/memory-like phenotype and contribute to innate immunity against experimental filarial infection
Source: Front Immunol. 2022 Sep 27;13:969340. doi: 10.3389/fimmu.2022.969340 (PMC9551455; doi:10.3389/fimmu.2022.969340)

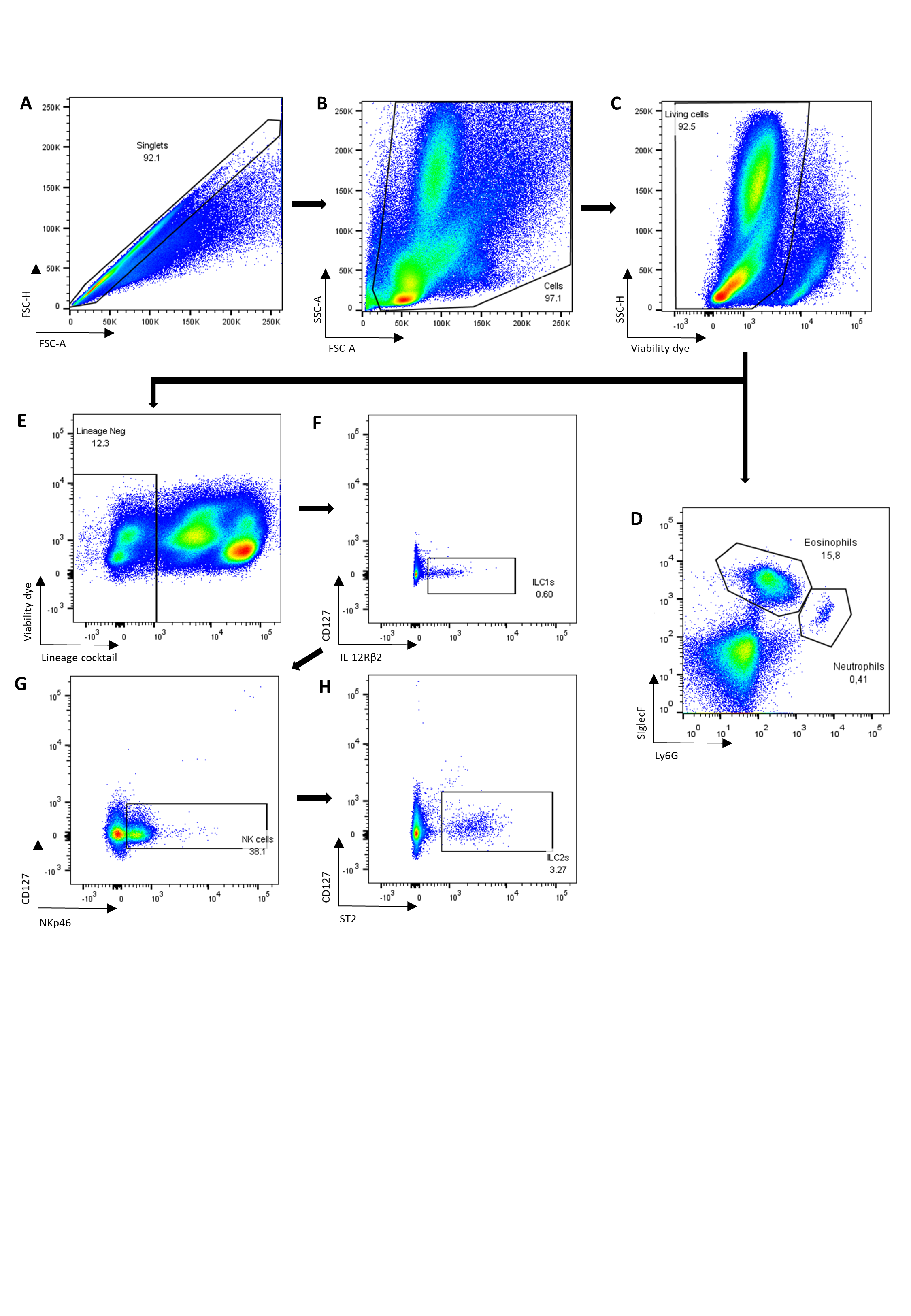

Supplement: Supplementary Figure 1 — Flow cytometry gating strategy for leucocytes immunophenotyping. Doublets (A), debris (B) and dead cells (C) were excluded and pan markers antibodies were utilised for the different cell populations. (D) Eosinophils and neutrophils were gated as SiglecF+ Ly6G- and SiglecF- Ly6G+ respectively. Innate lymphoid cells (E–H) were pre-gated on lineage negative cell populations (E) (CD8-, B220-, F4/80-, SiglecF-, CD4-, Ly6G-, FcγR1-) and identified as CD127-/lowIL-12Rβ2+ST2-NKp46- for ILC1s (F), CD127lowIL-12Rβ2-ST2+NKp46- for ILC2s (G) and CD127-/lowIL-12Rβ2-ST2-NKp46+ for NK cells (H). [file Image_1.tif]

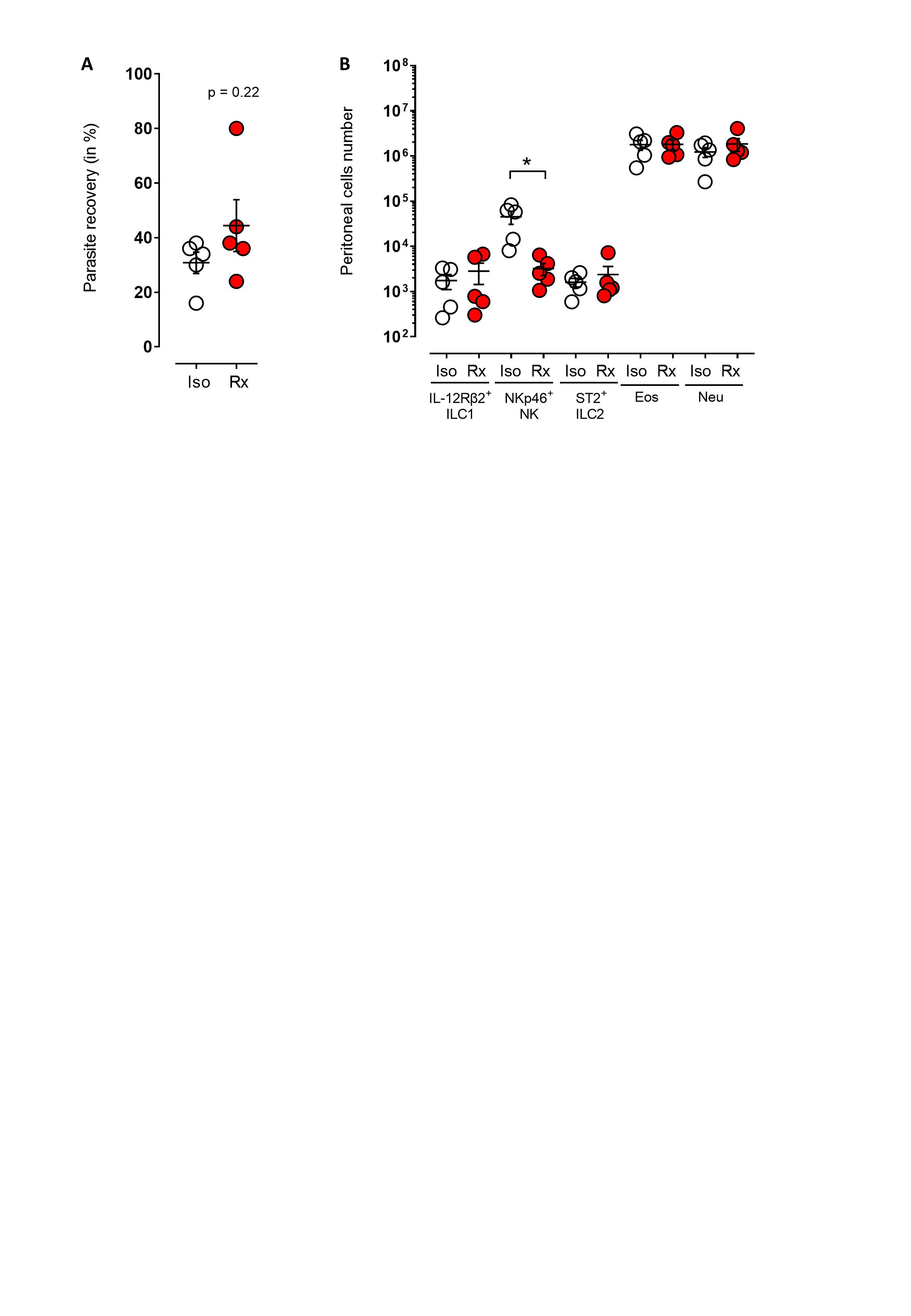

Supplement: Supplementary Figure 2 — Short-term temporal ablation of NKp46+ NK cells in RAG2-/- mice is not sufficient to confer treated mice an increased susceptibility to B. malayi parasites. B. malayi infected RAG2-/- mice were intraperitoneally injected with 0,5mg anti-NKp46 antibody or its isotype control in two occasions (at day 0 and day 3 post-infection) and culled at 1 week post-infection. (A) Parasite recovery in isotype control treated mice (Iso) or anti-NKp46 treated mice (Rx) at readout. (B) Innate lymphoid cells (IL-12Rβ2+ ILC1, NKp46+ NK, ST2+ ILC2) and granulocytes (eosinophils – Eos and neutrophils – Neu) numbers in the peritoneal cavity of control (Iso) or anti-NKp46 treated (Rx) infected mice at readout. Unpaired T-tests, n=5, single experiment. Significance is given as *: p <0.05. [file Image_2.tif]
